# Community Members’ Perceptions of a Resource-Rich Well-Being Website in California During the COVID-19 Pandemic: Qualitative Thematic Analysis

**DOI:** 10.2196/55517

**Published:** 2024-03-25

**Authors:** MarySue V Heilemann, Jianchao Lai, Madonna P Cadiz, Jocelyn I Meza, Daniela Flores Romero, Kenneth B Wells

**Affiliations:** 1 School of Nursing University of California, Los Angeles Los Angeles, CA United States; 2 Department of Social Welfare Luskin School of Public Affairs University of California, Los Angeles Los Angeles, CA United States; 3 Department of Psychiatry and Biobehavioral Sciences David Geffen School of Medicine University of California, Los Angeles Los Angeles, CA United States; 4 Research Center for Health Services and Society Jane and Terry Semel Institute for Neuroscience and Human Behavior University of California, Los Angeles Los Angeles, CA United States

**Keywords:** adaptation, humans, pandemics, mental health, COVID-19, health resources, California, psychological, stigma, digital, prevention, public health, emotions, website, qualitative research

## Abstract

**Background:**

To address needs for emotional well-being resources for Californians during the COVID-19 pandemic, the Together for Wellness/Juntos por Nuestro Bienestar (T4W/Juntos) website was developed in collaboration with multiple community partners across California, funded by the California Department of Health Care Services Behavioral Health Division federal emergency response.

**Objective:**

This qualitative study was designed to explore and describe the perspectives of participants affiliated with California organizations on the T4W/Juntos website, understand their needs for web-based emotional health resources, and inform iterative website development.

**Methods:**

After providing informed consent and reviewing the website, telephone interviews were conducted with 29 participants (n=21, 72% in English and n=8, 28% in Spanish) recruited by partnering community agencies (October 2021-February 2022). A 6-phase thematic analysis was conducted, enhanced using grounded theory techniques. The investigators wrote reflexive memos and performed line-by-line coding of 12 transcripts. Comparative analyses led to the identification of 15 overarching codes. The ATLAS.ti Web software (ATLAS.ti Scientific Software Development GmbH) was used to mark all 29 transcripts using these codes. After examining the data grouped by codes, comparative analyses led to the identification of main themes, each with a central organizing concept.

**Results:**

Four main themes were identified: (1) having to change my coping due to the pandemic, (2) confronting a context of shifting perceptions of mental health stigma among diverse groups, (3) “Feels like home”—experiencing a sense of inclusivity and belonging in T4W/Juntos, and (4) “It’s a one-stop-shop”—judging T4W/Juntos to be a desirable and useful website. Overall, the T4W/Juntos website communicated support and community to this sample during the pandemic. Participants shared suggestions for website improvement, including adding a *back* button and a drop-down menu to improve functionality as well as resources tailored to the needs of groups such as older adults; adolescents; the lesbian, gay, bisexual, transgender, and queer community; police officers; and veterans.

**Conclusions:**

The qualitative findings from telephone interviews with this sample of community members and service providers in California suggest that, during the COVID-19 pandemic, the T4W/Juntos website was well received as a useful, accessible tool, with some concerns noted such as language sometimes being too “professional” or “clinical.” The look, feel, and content of the website were described as welcoming due to pictures, animations, and videos that showcased resources in a personal, colorful, and inviting way. Furthermore, the content was perceived as lacking the stigma typically attached to mental health, reflecting the commitment of the T4W/Juntos team. Unique features and diverse resources, including multiple languages, made the T4W/Juntos website a valuable resource, potentially informing dissemination. Future efforts to develop mental health websites should consider engaging a diverse sample of potential users to understand how to tailor messages to specific communities and help reduce stigma.

## Introduction

### Background

The COVID-19 pandemic brought unexpected difficulties related to activities of daily living for people worldwide. In addition to affecting physical health, COVID-19 also threatened emotional health. Diverse groups were impacted, including people of different ages [1-4], gender identities [5-9], races and ethnicities [10,11], and geographic locations [12-15]. Those who faced financial concerns [16,17] or lost their jobs [8,18] and those caring for children at home [5] struggled with a variety of additional pressures. For workers [19,20], including health care workers [21,22] and community-based service providers [23-26] in the United States, attempts to serve clients or patients were confounded by pandemic-related challenges such as concerns about infection, reductions in staffing, and transitions to remote care, making their jobs even more complex and challenging.

As stay-at-home orders proliferated, people began to look for ways to strengthen their ability to cope emotionally during the pandemic. Many turned to media outlets such as television, radio, and social media for news and information [27]. Unfortunately, the news often led to increased fear and worries about the COVID-19 virus, illness, death, loss of jobs, economic concerns, and more. Many used Twitter to read the views of others and express their own negative sentiments about the pandemic [28]. Social media use contributed to experiences of stress [27], whereas engagement in self-care activities such as being able to access and use personal support resources helped protect against mental health distress [29]. Consequently, researchers [27,30] called for creative developments to connect individuals with social support and mental health services. To overcome stigma and other barriers, researchers and developers turned to web-based digital tools to make resources for coping and information on emotional well-being more accessible.

In this context, the Together for Wellness/Juntos por Nuestro Bienestar (T4W/Juntos) website was developed in collaboration with multiple community partners across California, funded by the California Department of Health Care Services Behavioral Health Division federal emergency response, to directly address needs for free web-based emotional well-being resources for Californians during the pandemic. The purpose of this paper is to report the findings of a qualitative study on the perspectives of a sample of participants affiliated with California organizations who engaged with the T4W/Juntos website.

### Development of the T4W/Juntos Website

The T4W/Juntos website was developed as part of the Federal Emergency Management Agency and Substance Abuse and Mental Health Services Administration crisis counseling contract with California. The goals for T4W/Juntos were developed with a multidisciplinary team of researchers, clinicians, digital resource development experts, and staff from community-based agencies in California. Goals centered on creating inclusive and accessible resources that would provide evidence-informed and evidence-based information to Californians to ease the stress experienced during the pandemic [31].

Meetings were held via Zoom (Zoom Video Communications) with the large collaborative team (4 to 18 members per meeting) to maximize input from community members. The community and study team members made decisions collaboratively about which types of resources to include on the website. The priority was to feature resources that facilitated learning about COVID-19 or offered ways to address anxiety and stress (eg, web-based meditations, breathing exercises, and direct links to warmlines and hotlines), strengthen resilience, cope with grief due to a recent loss, connect with other people (such as through web-based support groups), or support social justice (eg, antiracism and reducing hate crime). Resources included links to web-based toolkits, websites, videos, web-based applications, articles, and downloadable pamphlets. Some resources were available in multiple (up to 10) languages. Community partners’ emphasis on using neutral, nonclinical language to increase comprehension and relatability and reduce stigma led to a monitoring of the length and complexity of messaging for the website. In response to the community partner prioritization of videos to engage users, the team created videos of community members speaking about the website’s purpose and features in English and Spanish [31].

### Prior Work

A previous paper related to T4W/Juntos described the process of website development [31]. A second paper described the results of an analysis of quantitative data from an electronically administered web-based survey that were collected at 2 time points (approximately 6 wk apart) from English- and Spanish-speaking adult participants. Of the 366 eligible participants, 315 (86.1%) completed the baseline survey and 193 (61.3%) completed the follow-up survey, with baseline results showing substantial diversity in gender, gender identity, and race and ethnicity and 32.7% (103/315) having moderate depression or anxiety (2-item Patient Health Questionnaire or 2-item Generalized Anxiety Disorder score of ≥3) [32,33]. Significant predictors of baseline website engagement were Hispanic versus other race or ethnicity and COVID-19–related behavior changes. The use of the T4W/Juntos website during the month before the follow-up survey was significantly associated with a pretest-posttest reduction in depression (2-item Patient Health Questionnaire score), and greater website engagement at baseline predicted reduced hotline use before follow-up [34]. An analysis of short qualitative answers that 199 (63.2%) out of 315 participants typed into textboxes in response to open-ended questions in the previously described web-based survey led to insights into safety concerns and fears during the pandemic and perceived benefits from and suggestions for improving the website [35].

### Research Aims

With the goal of supplementing the quantitative results, the aim of this qualitative study was to describe the perspectives of a diverse subset of participants associated with various California community organizations who completed the baseline surveys regarding their experiences with the T4W/Juntos website. We also focused on participants’ needs for website resources that could support emotional well-being for themselves, their families, their clients, or their community. Finally, we sought insight to inform iterative website development in the future.

## Methods

### Recruitment

The larger sample described previously was recruited during the pandemic through invitations that were sent primarily by email, while stay-at-home orders were in effect, from 11 community partner agencies throughout the state of California to their affiliated community members with information about the website, its purpose, and the research study. Each of the 315 participants who consented and completed the baseline survey was given the option to indicate their interest in participating in a potential future telephone interview by clicking a box at the end of the survey. In total, 73.9% (233/315) of the participants clicked on the box to indicate their interest in being interviewed. Inclusion criteria were being aged ≥18 years, having access to the internet, having already completed the baseline web-based survey in English or Spanish, and agreeing to provide contact information. Using convenience sampling, participants who spoke English or Spanish were contacted via telephone by research staff approximately 2 weeks after completion of the baseline survey, starting with those who were the first to finish the survey, to offer an interview, confirm availability, and set a date and time for the interview. After 15 interviews were conducted, purposive sampling was used to maximize diversity in race and ethnicity, gender, and age. The final sample comprised 29 participants. The interviews (in English or Spanish) were conducted between October 2021 and February 2022.

### Ethical Considerations

This study was reviewed and approved by the University of California, Los Angeles, Institutional Review Board of UCLA’s Human Research Protection Program (20-002163-AM-00008). After reading the web-based consent document, each participant clicked to give consent at the time of enrollment in the larger survey study, which included consent to a future potential interview. Our team only contacted individuals who agreed to be contacted for interviews using the contact information they provided. Participants reconfirmed their approval to participate and be audio recorded at the time of the interview. To protect the privacy and confidentiality of participants, the list of the names of the participants and their assigned codes was kept in a password-protected file available only to the principal investigator and project director. Their confidential contact and personal information were kept separate from all other data. Any potentially identifying information was deidentified on the transcriptions of audio-recorded interviews, including any names or descriptors that could possibly identify a participant; all names were changed to code numbers that were used instead of names by the researchers during data analysis. Participants received a US $25 e-gift card after completing the interview.

### Data Collection

Demographic data were retrieved from the baseline survey for each of the 29 interview participants. A semistructured interview guide in English and Spanish that was developed by a multidisciplinary team was subsequently used by 2 research team members to conduct all interviews via telephone. Interview questions were designed to explore participants’ perceptions of any aspect of the T4W/Juntos website; gain insight into participants’ needs for support in relation to the resources available via the website for themselves and their families, clients, or communities; and obtain guidance on further development of the website. Audio recordings of interviews in Spanish were professionally translated into English, and all interviews were professionally transcribed verbatim and checked for accuracy. As already noted, identifiers were removed, and code numbers were used instead of names to label transcripts and organize the data.

### Data Analysis

Demographic data were analyzed for frequencies using Stata/MP (version 17; StataCorp LLC) [36] for the sample of 29 participants. For the thematic analysis of the 29 transcripts, the study team was guided by a modification of the 6-phase process outlined by Braun and Clarke [37,38]. First, the study team familiarized themselves with the data in all transcripts. Second, the team engaged in initial coding using techniques from grounded theory methodology to enrich our approach [39]. Thus, most codes were developed using the gerund form of verbs, known as process codes, to heighten our focus on the actions taken by participants, as shown in the data [39,40]. To create process codes, coders used heuristic questions to ask *What is happening here?* and *What are they doing here?* This allowed coders to get closer to the participants’ point of view while reducing the tendency to prematurely project their own interpretations onto the data [39]. In the third and fourth phases, coders scrutinized the first 12 coded transcripts to identify the most frequently occurring and significant codes and, through discussion and debate, identified a total of 15 overarching codes. Then, the data from all 29 transcripts were imported into ATLAS.ti Web (version 22.1.5; ATLAS.ti Scientific Software Development GmbH) [41] and coded based on the 15 overarching codes. In the fifth phase of analysis, data reports were created using ATLAS.ti Web [41] based on each of the 15 overarching codes. These were exported to Microsoft Excel (Microsoft Corp) so that the data in each code group could be further examined. Using constant comparison, we sifted, sorted, combined, and collapsed the data in the 15 groupings to form 4 themes, each with a central organizing concept that provided a clear definition of the theme [38]. We continued to compare data with data to develop the properties for each of the 4 themes. Finally, in the sixth phase, each theme was named, and its properties were refined. With a focus on the research aims, the research team then produced a written report interpreting the meaning of each theme.

The overall process of data collection and analysis was influenced by the team’s commitment to social justice and to the goal of understanding the data of each participant while considering their context. Thus, at various points during the research process, each member of the 5-member analysis team engaged in dialogue together and in individual writing of reflexive memos to name any judgments (positive and negative) or concerns that were felt while engaged in the research process, with the goal of reducing the influence of bias on the collection and interpretation of data [38,39].

## Results

### Participant Characteristics and Sample Demographics

The demographics of our sample of 29 participants are presented in Table 1. Of the 29 participants, 16 (55%) voluntarily shared that they were employed in peer support, hospice care, or health care sales or at a community agency doing health-related work. A total of 72% (21/29) of the interviews were conducted in English, and 28% (8/29) were conducted in Spanish. The duration of the Spanish interviews ranged from 22 to 72 (mean 32, SD 16.61) minutes, and that of the English interviews ranged from 15 to 85 (mean 38, SD 15.49) minutes.

**Table 1:** Demographics and depression and anxiety scores of community participants in California who were interviewed during the COVID-19 pandemic (N=29).

Characteristic			Values
**Language, n (%)**			
	English	21 (72)	
	Spanish	8 (28)	
Age (y), mean (SD)			46.3 (13.7)
**Education, n (%)**			
	Some high school or lower than high school	2 (7)	
	High school graduate or equivalent (ie, GED^a^)	1 (3)	
	Some college	9 (31)	
	College graduate	12 (41)	
	Graduate school (eg, JD^b^, Master’s, PhD^c^, and MD^d^)	5 (17)	
**Gender, n (%)**			
	Woman	20 (69)	
	Man	7 (24)	
	Other gender not listed (2-spirit)	1 (3)	
	Prefer not to state	1 (3)	
**Race and ethnicity, n (%)**			
	American Indian or Alaska Native	1 (3)	
	Black or African American or African	5 (17)	
	Hispanic or Latino (Cuban, Mexican, Puerto Rican, South or Central American, or other Spanish culture or origin regardless of race)	10 (34)	
	Southeast Asian (Vietnamese, Filipino, Laotian, Thai, Indonesian, and Cambodian)	2 (7)	
	White or European	7 (24)	
	Multiracial	3 (10)	
	Unknown	1 (3)	
**Sexual orientation, n (%)**			
	Straight	21 (72)	
	Gay or lesbian	1 (3)	
	Bisexual or pansexual	2 (7)	
	Other sexual orientation not listed	1 (3)	
**GAD-2^e^, n (%)**			
	0-4 (none to minimal)	27 (93)	
	5-9 (mild)	2 (7)	
**PHQ-2^f^, n (%)**			
	0-4 (none to minimal)	26 (90)	
	5-9 (mild)	3 (10)	

^a^GED: General Educational Development.

^b^JD: Juris Doctor.

^c^PhD: Doctor of Philosophy.

^d^MD: Medical Doctor.

^e^GAD-2: 2-item Generalized Anxiety Disorder scale. A GAD-2 score of 3 is the recommended cutoff point for identifying possible cases of generalized anxiety disorder.

^f^PHQ-2: Patient Health Questionnaire–2. A PHQ-2 score of 3 is the recommended cutoff point for identifying possible cases of depression.

### Qualitative Thematic Analysis Results

Thematic analysis of the qualitative data led to the identification of four themes: (1) having to change my coping due to the pandemic, (2) confronting shifting perceptions of diverse groups on mental health stigma, (3) “Feels like home”—experiencing a sense of inclusivity and belonging in T4W/Juntos, and (4) “It’s a one-stop-shop”—judging T4W/Juntos to be a desirable and useful website.

#### Theme 1: Having to Change My Coping Due to the Pandemic

##### Overview

**Figure 1:**
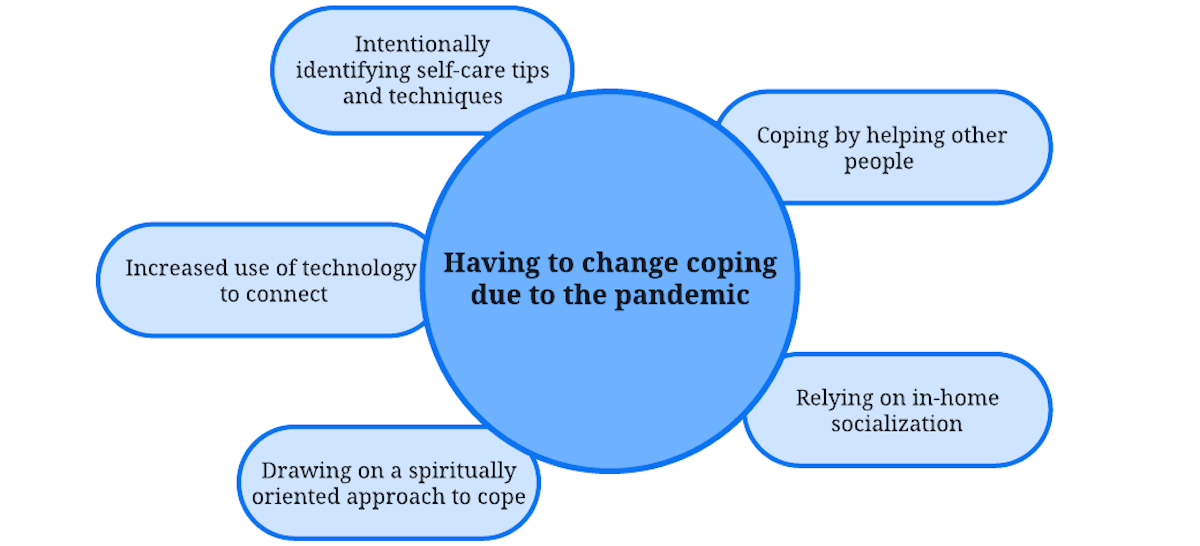
Theme 1 from qualitative analysis of interview data from community participants in California during the COVID-19 pandemic (N=29): having to change my coping due to the pandemic.

Participants shared that, during the pandemic, they had to change the way in which they coped with daily life stressors. This was represented by 5 properties: increased use of technology to connect with others on the internet, intentionally identifying self-care tips and techniques, coping by helping other people, relying on in-home socialization, and drawing on spiritually oriented coping (Figure 1).

##### Increased Use of Technology to Connect

Participants found that their use of technology increased during the pandemic, and they had to learn to accept their increased reliance on the internet to be connected in various ways. For example, they used technology to connect with information on a variety of topics and be able to accomplish work for their jobs. They used technology to connect with other people for social reasons; this included using Zoom to connect with friends, family, and their faith communities. They also joined web-based support groups and community groups where they could engage in dialogue with others. One participant described how they felt more “comfortable” having conversations on the internet:

I think I’ve become more dependent on the internet. I also found that not having to deal with people face to face most of the time makes me feel more comfortable. Honestly, it’s easier for me to have a chat on the computer than in real life.

Participants said that they relied on technology to meet their therapeutic needs more than before the pandemic. They found web-based resources to be “easier to forward” and share with others. They described resources on the internet to be “more documented” and viewable. They reported how they learned to click links to use web-based resources, which, for many, was a new behavior.

##### Intentionally Identifying Self-Care Tips and Techniques

Participants helped themselves by seeking out practical approaches, including self-care tips and techniques. This meant that they were using technology to meet their therapeutic needs, something they had not necessarily done before. They used warmlines, crisis lines, and teletherapy to meet their needs. They learned about meditation and breathing exercises, which they found especially desirable because the stress of the pandemic was experienced as personally difficult. However, participants found it challenging to find “accurate” resources. This put them on a quest to find “reliable” web-based resources they could use to reduce stress. Participants explained that they continued searching on the internet even if their immediate need was resolved because they wanted to have resources ready just in case they needed them in the future. In addition, some used art to self-soothe during the pandemic, whereas others sought “self-improvement” strategies.

##### Coping by Helping Other People

When asked to say more about how they handled their own stressful experiences during the pandemic, participants repeatedly spoke of helping other people in their personal lives and on the job. It seemed that helping other people was itself a strategy they used to cope. A participant spoke of others who felt “invisible” and as if other “people don’t respect them” and how difficult it was for them because “they’ve lost their purpose because they can’t go to work or can’t do the job they used to” do. They noted how important it was to share with others that “there’s hope...that there are people out there trying to make a difference, trying to help, trying to listen*.*” Participants’ efforts were extended to various types of people, including family members, friends, and coworkers, and those who self-identified as health workers reported that they helped both individual clients and families. Experiences of helping others stood out to them; they felt a sense of “satisfaction” from their helping work. One participant said the following:

And the reason I liked it is—and the reason is the feedback, the participation and everything, is actually one of the ways that it makes me feel like not only I’m sharing a resource, but I’m sharing a resource that I know is good—I hate sharing things that I know are not good—and I wanted to like what I’m doing here.

##### Relying on in-Home Socialization

Finding others to fulfill social needs and desires was mainly limited to whoever was in the home. Participants relied on their family members or roommates for dialogue, socialization, and friendship during the pandemic. However, pets also played an important role as they provided “joy, stress relief, and companionship.” Many described the importance of going on walks with their dogs as it brought about daily exercise and also could open up dialogue with neighbors, which was highly valued at this time of social isolation. One participant shared how meaningful it was to live with their 2 dogs and son. They said the following:

If I had to live by myself, I don’t know how I would get through this. I really mean it. I’m being perfectly honest.

##### Drawing on a Spiritually Oriented Approach to Cope

With few options for socialization, participants shared that they turned to spiritually oriented routes for coping. They turned to “God” and relied on their faith to keep them going. One participant stated that they could not imagine how anyone could “get through” the pandemic “without God.” For some, listening to religious radio programs filled a crucial need in their daily lives during the pandemic. Others reported using prayer or reading scriptures. A participant described how religion provided “guidance” on daily life:

[Faith in God] helps me first and foremost, that helps me not to have fear. I think that a lot of people now are controlled by fear. And so that helps me, y’know; that strengthens me and gives peace to my heart. I feel secure with my health habits, with my diet, well, because I’m connected to God, and because I get my health practices from the Bible. So, I feel that all around, my mental, my physical, my emotional, all my wellbeing, I dedicate that to God for his blessing. And so, y’know, I think that’s the biggest part of it.

#### Theme 2: Confronting a Context of Shifting Perceptions of Mental Health Stigma Among Diverse Groups

##### Overview

**Figure 2:**
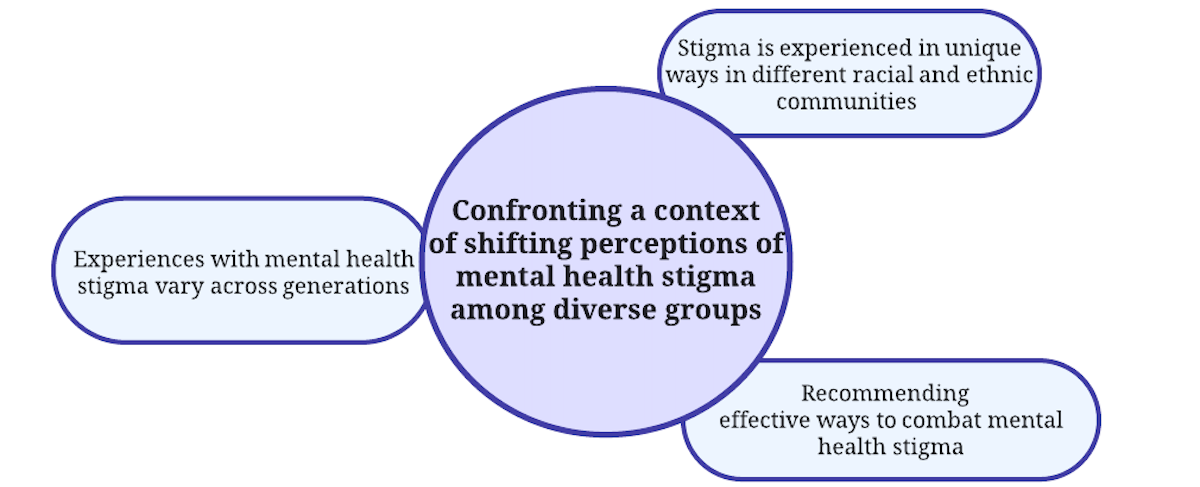
Theme 2 from qualitative analysis of interview data from community participants in California during the COVID-19 pandemic (N=29): confronting a context of shifting perceptions of mental health stigma among diverse groups.

When discussing T4W/Juntos and its purpose in helping with emotional well-being, participants were concerned about the context during the pandemic related to public views on mental health. They raised the issue of shifting views on mental health stigma within different communities and how it impacts people. They reported how stigma differs based on age or generation, race, or ethnicity and how the T4W/Juntos website would be received within a context of overarching stigma in various communities. They also suggested ways to reduce stigma. This is reflected in 3 properties (Figure 2).

##### Experiences With Mental Health Stigma Vary Across Generations

Mental health stigma was perceived as an issue for all generations. Participants explained that stigma itself was the backdrop that set the stage for how the T4W/Juntos website would or would not be received. This could have an impact on whether community members would embrace the website.

Participants explained that there was a difference in how older and younger generations experienced mental health stigma. Older adults who grew up during a time when mental illness was considered “bad” and “dangerous” were described as rejecting the possibility of seeking treatment for their mental health problems. They described a dual process in which the older generation felt too stigmatized to seek mental health support, but at the same time, they perpetuated the stigma surrounding mental health within their communities. Older adults were perceived as having negative beliefs about mental health concerns, sometimes viewing them as a personal “weakness” or a result of lack of religious practice (eg, “devil’s work”) rather than a psychological condition. They were seen as discouraging other people from reaching out for such treatment, help, or resources. One participant shared how older people spoke about mental health treatment:

*Looking for help, like, with a therapist...they shouldn’t be sharing their opinions.* *That’s what I’ve heard, “Why would you see a therapist, if they’re not God?”...that’s* *what I’ve heard. Like, “Why should you go around telling them your problems?”*

Isolation was perceived as a major contributor to older adults’ mental health issues, especially for those who lived in residential settings. This raised concerns during the pandemic because participants reported that resources tailored to older adults living in such settings were not available, especially for those who were “losing loved ones” due to COVID-19. Participants worried about older adults “not being able to see their families” during the COVID-19 pandemic and how they would cope as stigma could be a barrier to obtaining the help they needed.

Younger generations were perceived as having grown up with greater awareness of mental health and, therefore, were affected by stigma in a different way. They were thought to be “more empowered” to openly discuss mental health issues. In particular, participants noted a more welcoming conversation about mental health on social media among younger generations, including during the pandemic. The differences between generations were described by one participant as follows:

*I feel like that [the older generation] was like, “We’re not telling anyone our business.”* *And “This is family business, keep it to yourself.” Whereas like the mid-30s and maybe* *late 20s, they’re like, “You know what? Let’s talk to somebody, let’s get help, let’s like—We’re not going to suffer in silence.”*

##### Stigma Is Experienced in Unique Ways in Different Racial and Ethnic Communities

Mental health stigma was perceived as experienced variably based on race and ethnicity, which had implications for the context of the pandemic. Participants shared examples of how Black, Latine, Asian, and other minoritized communities faced more mental health stigma in general compared with other communities due to a combination of societal and cultural factors. One Latine participant said the following:

*Because I grew up culturally Latino, so, there is a huge stigma around mental health* *where you couldn’t just say, “Oh, I’m feeling anxious,” or “I’m feeling a little* *depressed.”*

Another participant said that, in their Asian American community, mental health was “heavily stigmatized” and people “don’t tend to like to ask for help.”

The words participants heard being used to stigmatize mental health or people with emotional challenges, such as “crazy” and “weak,” were similar across racial and ethnic minoritized groups. While all people were seen as actively avoiding being labeled as having a mental health issue, those from minoritized communities were perceived as especially cautious because such negative mental health labels could be used as “leverage” against them. Therefore, a winning strategy used by community-based health workers in our sample was to “give them the information without having to use that word [mental health].” Others described avoiding being labeled by addressing physical rather than psychological symptoms. This provided a perspective for addressing mental health by taking care of physical health. One participant explained this as follows:

I think if the focus is not so much on mental illness but mental wellness, mental health, and that connection between the mind and the body, and that it’s all important, and addresses the person as a whole.

##### Recommending Effective Ways to Combat Mental Health Stigma

Participants recommended providing accessible educational resources on mental health to the public. One suggested that stigma could be reduced if mental health was discussed in the same way in which health providers engaged in “teaching someone how cancer works*.*” Other suggestions included the strategy of individuals openly sharing their personal stories of mental health struggles to dismantle stigma and encourage help-seeking behaviors. One participant explained that we need “to recognize that we all have trauma, and to set the example ourselves.”

In terms of sharing the T4W/Juntos website and other resources, participants suggested that, rather than just directing people where to go, we should share our experiences honestly. One participant recommended saying things such as “This happened to me and I went here to get help. That helped me a lot because I did this” or “I also went through the same situation.”

Participants encouraged efforts to create safe and supportive spaces, especially for marginalized individuals who may face additional stressors, such as “LGBTQAI+ students,” adolescents, and older adults. Participants reported that they would feel more comfortable recommending a warmline where individuals could connect with trained volunteers, therapists, or peers who could listen and provide support rather than “an Excel [sheet] of resources.” While participants endorsed efforts such as the T4W/Juntos website, they recommended investing in “more intersectional conversations” where leaders “that really represent a community” could share things such as “this is me, and this is what I’m going through. This is what I do to deal with it. This is where I go for help*.*” This approach was perceived to increase “comfort” and “acceptance,” unlike “faceless” things, because it could reduce stigma and negative perceptions of help seeking.

#### Theme 3 “Feels Like Home”: Experiencing a Sense of Inclusivity and Belonging in T4W/Juntos

##### Overview

**Figure 3:**
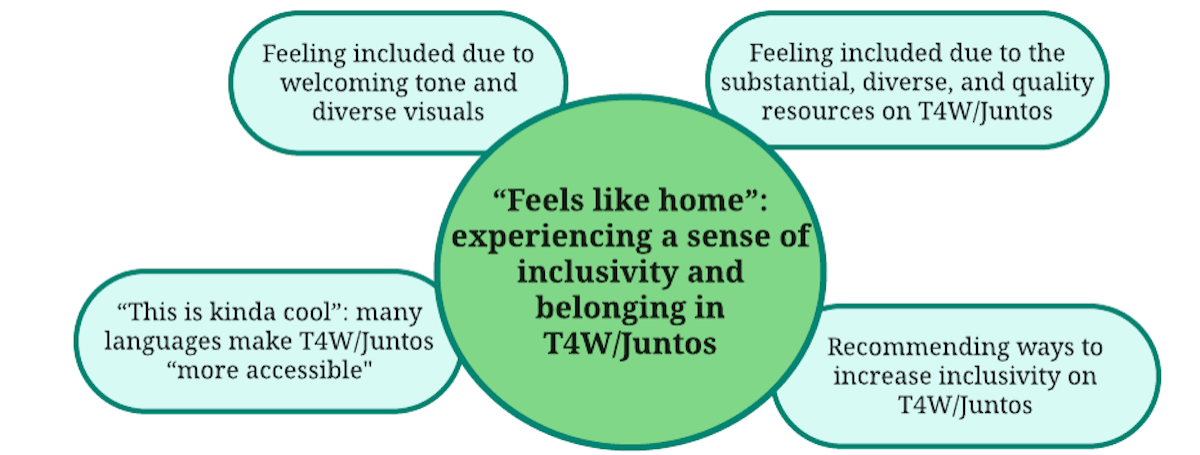
Theme 3 from qualitative analysis of interview data from community participants in California during the COVID-19 pandemic (N=29): “Feels like home”—experiencing a sense of inclusivity and belonging in Together for Wellness/Juntos por Nuestro Bienestar (T4W/Juntos).

Participants provided robust reports of feeling welcomed and included when visiting the T4W/Juntos website. This sentiment is reflected in 4 properties: feeling included due to welcoming tone and visuals; feeling included due to the substantial, diverse, and quality resources on T4W/Juntos; many languages making T4W/Juntos “more accessible”; and recommending ways to increase inclusivity on T4W/Juntos (Figure 3).

##### Feeling Included Due to Welcoming Tone and Diverse Visuals

The images and overall tone of T4W/Juntos gave participants a sense of belonging when using the website. They described the site’s imagery as “cheerful,” “friendly,” “bright,” “happy,” “fun,” “calming,” and “light and airy.” One participant explained the following:

...the color scheme and the font, it’s just very inviting and not intimidating. ‘Cause I think finding like health resources or mental health services or any of these topics, they’re very heavy. So, having a page that’s bright and makes it simple and has the cute little icon next to each topic makes it a little more digestible.

Other participants focused on T4W/Juntos’ esthetics. One participant indicated that it was “well-balanced” with “just enough seriousness.” Another participant highlighted the site’s “high production value” in terms of visual and auditory content. The quality of the content was valued, including the mix of both cartoon and real images, the “scenery” in graphics, and the quality of the spoken Spanish in videos.

Participants noted the importance of diversity in T4W/Juntos’ images in helping them feel included. They appreciated seeing the “authentic representation” of various ages, gender identities, abilities, sexualities, and races and ethnicities, among other characteristics, in “these beautiful faces” they saw on the website. Participants indicated that T4W/Juntos “feels like home” because the images were specifically representative of California’s population. One participant stated the following:

I feel like it covered populations and community members across California who would be possibly using the website. And also, just showing that diversity. So, I think that creates a welcoming environment as well if people can see themselves represented in some capacity on the website, especially on the front page. 

Unlike other mental health websites that participants described as judgmental or exclusionary, participants felt that T4W/Juntos was not overly “clinical” or “bashing you with some mental illness stigma.” Furthermore, T4W/Juntos’ diverse representation was different from that of other sites where “only one type of person” was represented, which meant that visitors to T4W/Juntos would not “feel like they’re an outsider,” as one participant succinctly explained:

*...if there’s nobody on the website that I can identify with, maybe it doesn’t...doesn’tapply to me kind of thing. There’s plenty of opportunity, I think, for anyone to feel likethey fit [on T4W*/Juntos*].*

##### Feeling Included Due to the Substantial, Diverse, and Quality Resources on T4W/Juntos

The quality, quantity, and variety of resources present on the T4W/Juntos site greatly contributed to participants feeling welcome and included. Some expressed appreciation in broad strokes, noting that there were resources for “every ethnicity” and “different ages” and that the T4W/Juntos team “took many different things into account.” Other participants valued the inclusion of resources for specific groups, such as African American individuals, American Indian and Alaska Native individuals, the “LGBTQIA2+ community,” parents, children, and people living with disabilities. Knowing that the resources were *intentionally* selected for T4W/Juntos mattered. One participant noted that “...the thought put into making [T4W/Juntos] usable or worthwhile to a number of different communities was made and paid attention to*.*” Another interviewee found it “refreshing to realize that the [T4W/Juntos] project...had equity kinda built from the top up.” One participant encapsulated this by saying the following:

It just felt like I was coming to a buffet, a big place to finally like heal, y’know? It was like “Oh, I don’t even have to eat this. There’s like a little bit of that, more of that.” And there’s really—the variety of choices, and the way that it was put, it was very inviting. Also, it was very welcoming, and I left feeling satisfied, but also, I left—like there was stuff that I could share with people. And I did.

##### “This Is Kinda Cool”: Many Languages Make T4W/Juntos “More Accessible”

Linguistic accessibility was another important aspect of the T4W/Juntos site that made participants feel included. Because of California’s multicultural population, participants believed that T4W/Juntos needed to be offered in multiple languages, especially Spanish and Vietnamese, for it to be considered “culturally appropriate” and widely accessible. One participant stated that “I love that the website already has a few options in different languages...Just thinking about the different audiences [will] make it that much more accessible*.*” The creation and availability of a Spanish translation contributed to another participant’s feelings of inclusion:

So, it made me welcome and then it also made me understand more, like I said, because it was in Spanish and English.

Others noted the need for content in more languages; some specified a desire for content in Vietnamese, Indigenous languages (broadly), and Mixtec due to the large population of Mixtec-speaking migrant workers in California.

In addition to being offered in multiple languages, many participants lauded the site for being written in “everyday” and “plain” language that was “easy to understand” and “basic.” Another participant expressed their thought process upon first hearing about T4W/Juntos:

*I was like, “This is kinda cool. Let’s check this out.” It wasn’t something like, “Oh, wait,* *this is way beyond my expertise, or this is something I don’t fully understand.”*

Although many found T4W/Juntos easy to understand, a few respondents said that the language was inaccessible or overly professional. For instance, one person commented that the “writing [in T4W/Juntos] was too academic,” and therefore, it “wasn’t an easy read. It was like reading a textbook or a law book*.*” Others noted that terms such as “resilience” or “anxiety” made the site feel overly clinical and not intended for the average user.

There was concern that some users with low computer literacy may not be able to use the site. For example, one participant cautioned that some Spanish speakers may not necessarily know the word for “link” in English or Spanish (*enlace*). Furthermore, several participants who worked with immigrant communities indicated that many in these groups cannot read or write in Spanish or English, which precludes them from making use of the T4W/Juntos site.

##### Recommending Ways to Increase Inclusivity on T4W/Juntos

Some participants advocated for changes or additions that would further widen the net of inclusivity. For example, participants suggested adding information on mental health symptoms and treatment options; trauma and its potential effects; support for basic needs such as housing, rental assistance, and financial support; information about civic engagement (eg, how to register to vote and contacting local officials); and recreational activities such as art classes and book clubs.

Although many participants found the wide breadth of representation on T4W/Juntos to be quite impressive, some wanted even more diverse visual representation. Some perceived a few groups to be conspicuously absent on the site, such as the lack of representation of older adults. One participant stated the following:

...older adults have really had a hard time with isolation and access and I don’t really see older adults represented, at all—at all, at all, at all, like, at all, in this whole thing. Not just the graphics, but the people you’ve chosen to be on the little videos, the images, the content, there’s nothing about older adults, that I found.

Another participant specified the need for more youth representation:

*...make sure that we have as many opportunities for youth to be able to see themselves* *talking, working with each other, reaching out, but really knowing that we’re all here for* *them. And I think that was one of the things...that I would really suggest, is that* *opportunity.*

Other suggestions included adding more representation of men of color, including “African American men, Latino men and Armenian men,” as well as police officers and military veterans as these groups tend to avoid seeking treatment due to stigma against mental health. Others suggested that representation be enhanced with more images or voices of people with disabilities; individuals from the lesbian, gay, bisexual, transgender, and queer community; and actual community members.

#### Theme 4 “It’s a One-Stop-Shop”: Judging T4W/Juntos to Be a Desirable and Useful Website

##### Overview

**Figure 4:**
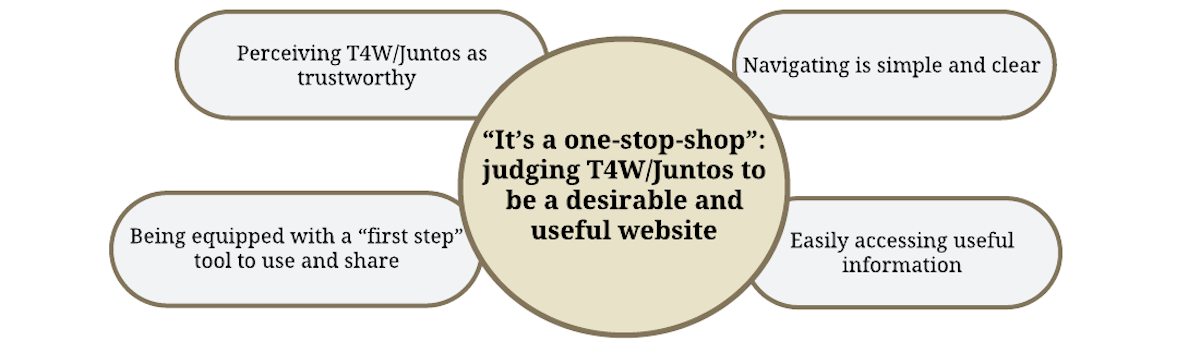
Theme 4 from qualitative analysis of interview data from community participants in California during the COVID-19 pandemic (N=29): “It’s a one-stop-shop”—judging Together for Wellness/Juntos por Nuestro Bienestar (T4W/Juntos) to be a desirable and useful website.

Participants judged the website as a hub that brought many things together in one place, making it a “one-stop-shop.” For this reason, most described it as a desirable website. Their perceptions reflect 4 main ways in which they experienced the website: perceiving T4W/Juntos as trustworthy, being equipped with a “first step” tool to use and share, finding navigation to be simple and clear, and easily accessing useful information (Figure 4).

##### Perceiving T4W/Juntos as Trustworthy

Participants valued the website due to the reliable information on it that had been curated from credible sources, which was able to combat misinformation. One participant reported the following:

*I feel like it’s done by UCLA, UC Davis, all names that I really trust...it’s a name that* *people recognize and that you can trust. So, I have no reservations whatsoever about this* *website...I know that when I scroll all the way at the end with the different sponsors or collaborations made it legit.*

Seeing the real faces and hearing the real voices of community members in the videos plus the logos of respected agencies on the website enhanced the sense that it was “legitimate.” Trustworthy information was especially desirable to combat the misinformation they reported hearing about in many communities.

##### Being Equipped With a “First Step” Tool to Use and Share

Participants felt equipped because the website gave them tools necessary not just for learning about resources but also for sharing with clients, friends, and family members. One participant noted how T4W/Juntos was something they had been searching for but had not found:

*And it just was primarily what it felt like, a resource portal. And so, it felt like the right* *door to go get help, rather than the wrong door. So that’s a good way of putting it.* *It felt like I had finally opened the right door that I’d been looking for.*

As a tool, T4W/Juntos was judged as helpful because it allowed participants to gain access to needed mental health resources during the pandemic, with options so that clients could start “where they want to” with the goal of receiving help. They liked having access to specialized information that addressed a wide range of topics, such as resources on grief for pregnant women or up-to-date COVID-19 information. They found it desirable because it was designed for the “average person,” so it was useful as a “first step” even for those with no previous knowledge about mental health resources.

##### Navigating Is Simple and Clear

Participants perceived T4W/Juntos to have appealing features such as inviting colors and fonts, which made the content “more digestible.” The technology functioned smoothly, including the hyperlinks and videos. One participant said the following:

*And it’s really easy to go on there, navigate, and look for information. And it’s also a* *good way to empower the clients I work with, so they can go and do their own* *research about any resources they may need in regards to mental health.*

The process of searching for information was clear even when working with groups lacking digital literacy, which they noted was required for some websites. One participant noted the following:

Honestly, this is one of the pages that I remember my mom and I—even though we were dazed and emotionally exhausted—we were able to understand and get the information because it was all so simple. That was the only thing I can tell you. Despite everything that happened, we saw that if we were going from one place to another looking for information or trying to analyze, sometimes it was very elaborate. We needed something like they say in English, “short and sweet,” not fancy or too negative. Something within the positive and informative things but without being research papers that we had to be reviewing and analyzing, because we didn’t have the capacity to do that. We needed simple and easy to understand information.

##### Easily Accessing Useful Information

The ease of using T4W/Juntos to access information was a valued feature. Participants found the website to be a user-friendly “one-stop-shop,” a place where they could find plenty of useful resources to choose from all at once. They preferred this to having to type specific topic words into a search engine to find needed resources individually. Some especially endorsed the feature that allowed them to receive immediate active help through a direct crisis number, whereas others favored the option to receive informational help by downloading materials to read. While participants overall considered T4W/Juntos to be easy to use, some suggested making the pages more “scroll-friendly...like Instagram,” and another participant suggested adding an “emergency exit” button so that users could quickly switch to a different site if needed for safety reasons.

## Discussion

### Principal Findings

The qualitative findings from telephone interviews as a complement to quantitative surveys suggest that, during the COVID-19 pandemic, the T4W/Juntos website was well received by both community health service providers and community members in the interview sample as a useful, accessible tool, with some concerns noted such as language sometimes being too “professional.” Our findings further suggest that the pandemic catalyzed significant changes in the way people coped, which fueled a shift to digital solutions when other options were suddenly off-limits due to stay-at-home orders. Our participants tended to their own emotional well-being personally and assisted their friends and families, and some also engaged in trying to help clients or patients as well. Their pivot to reliance on technology during the pandemic ranged from finding new techniques for soothing stress to connecting with others in a meaningful way via the internet to meet socialization and support needs. Roommates were crucial for socializing, as was also suggested by Shigeto et al [42], because social distancing limited social contact. Similar to other studies [43-45], our participants found pets to provide companionship and effective ways to cope with the isolation of stay-at-home orders. Notably, as other researchers found, being able to help other people during the pandemic in and of itself gave participants a mood boost [43,46]; this made the T4W/Juntos website even more valuable because participants could share it with others.

Similar to the findings of other studies [47-49], our participants shared that mental health stigma and taboo attitudes had often thwarted attempts to access needed mental health care, and this was especially the case for those from ethno-racially minoritized communities and older adults. However, participants did not perceive the T4W/Juntos site as invoking stigma, judgment, or condescension. They were particularly cognizant of the efforts of the development team to create a site that was neither intimidating nor shaming. They found it to be a digital space that successfully communicated that someone was out there trying to help others in a world that was otherwise shut down due to COVID-19. The collaborative approach to the development of the website may have been why the written text and verbal communication in the site’s videos were described as an example of a positive way to talk about mental health.

To combat stigma through a website, input from potential users, such as our participants, is crucial for design enhancement. As already noted, during the development of T4W/Juntos, input from various members of diverse California communities addressed the making of the website, including the goal of reducing stigma related to mental health [31]. Efforts to reduce stigma require sensitivity to the language used; with T4W/Juntos, we intentionally used neutral, nonclinical language so that experiences such as stress, anxiety, depression, and grief were addressed as normal aspects of life that many of us deal with [31]. Our participants recommended featuring pictures, animations, and videos to showcase resources in a personal way that is colorful and inviting without stigma and that reflects the commitment of the T4W/Juntos team. In addition, several short videos in English and Spanish were created to introduce each section of the T4W/Juntos website with the goal of making users feel more comfortable with the topics; volunteers from diverse California communities served as relatable actors who were filmed during the pandemic via Zoom in their homes. The diverse representation is likely why participants said that the website felt comfortable. We also included links to active warmlines and hotlines so communication with an actual human being was possible through telephone and texting [31,34,35]. However, consistent attention to making these links convenient and prominent on the website is needed over time to maintain a steady focus on reducing stigma. Additional efforts could be made in the future to link to more and different venues available on the internet where diverse community leaders talk about their own emotional health concerns or share what they have found helpful. In addition to the immediate sense of welcome, participants found the extent and variety of the content on T4W/Juntos (ie, the plentiful links to various resources) to communicate supply rather than unmet demand. The sense of options for resources was understood as high accessibility, which somehow also reduced stigma. Participants seemed to relish what they perceived to be a bounty of ready resource links, including content in multiple languages. This, during the pandemic, was appreciated because it was a time when avenues for the typical sources of useful or desirable material were severely reduced.

The sense of belonging reported by participants suggests the profound impact of a culturally competent design in enhancing user engagement with and experience on the website. For example, the colorful look of T4W/Juntos was developed in collaboration with community members [31]. The decisions to give T4W/Juntos an upbeat feel, feature relatable people from diverse California communities in the videos, and provide options in diverse languages were all made collaboratively with community partners. The team’s intentional efforts and commitment to convey diversity resonated with participants, making the website “feel like home.” Furthermore, as the partnering organizations behind the creation of the website were clearly listed on the site for the purpose of transparency regarding who was behind the website, participants perceived it to be a trustworthy tool.

Overall, our findings highlight that the T4W/Juntos website functions as a comprehensive, inclusive, and user-friendly platform for coping with mental health challenges, particularly during the pandemic. It was a web-based “one-stop-shop” due to the culmination of several integrated features that generated positive regard. However, despite its many strengths, there were also suggestions for improvements to the website to further enhance inclusivity. As was suggested by some participants, more resources, pictures, stories, and testimonials are needed to reduce stigma, specifically for older adults; adolescents; lesbian, gay, bisexual, transgender, and queer communities; police officers; and veterans. In terms of functionality, certain adjustments were requested, including a “back” button and a drop-down menu for a better user experience.

### Limitations

Some aspects of this study were restrained due to the pandemic. For example, we were only able to interview English- and Spanish-speaking adults. In addition, we used convenience sampling with recruitment based on invites from clients and community partners of staff and providers of community partner agencies. While this approach resulted in a diverse sample, it included community-based health and wellness workers and is not necessarily representative of California residents. Nonetheless, during the COVID-19 pandemic, these voices were extremely valuable and garnered important insights. While 55% (16/29) of the participants indicated that they worked in community-based support or health care, we did not collect specific data on employment status or occupation. We can only assume based on education and other factors that approximately half of our sample were community members not employed in health care. Thus, future research should systematically collect employment data as context for participants’ level of familiarity with health-related resources.

Relatedly, the COVID-19 pandemic put limits on potential participants’ ability to engage in a study when they were dealing with other worries. Thus, our sample was diverse in some ways but could have been more reflective of California’s population. For example, we were successful in recruiting 7% (2/29) of participants who were Southeast Asian; however, no participants self-identified as being from South Asian or East Asian communities despite the large numbers in California. Future research should expand recruitment efforts to be inclusive of the many subgroups in the state to bring insight from a more diverse sample.

### Conclusions and Future Implications

Our results complement the findings of the quantitative evaluation that showed engagement in website use and an association with reduced depression over time [34]. The results underscore the value of collaborating with members of the target community to have a meaningful impact when designing a digital tool for the public. Specific partner website design suggestions to include videos; language accessibility; diverse representation; and colorful, cheerful visuals contributed to the positive reception of this website. The findings suggest that, while T4W/Juntos has been effective in addressing diverse needs, there are ongoing opportunities to maximize inclusivity and user experience.

First, future studies on mental health website development would benefit from engaging with a diverse sample of the target group and conducting pilot tests to learn more specifically what accessibility means to potential users. Second, the results showed that mental health stigma continues to be an issue, especially among minoritized communities. Hence, resources tailored to such groups must consider what stigma looks like to members of each group and how to address it in the specific context of minoritized communities so that valuable information about mental health will be received and accepted. Finally, participants indicated that the T4W/Juntos website was useful for their personal needs, sharing with loved ones, or incorporating into their work with minoritized communities. The website’s unique features—especially its diverse representation and availability in multiple languages—make it a valuable addition to the mental health resource landscape, and thus, it may be recommended for dissemination throughout the state of California, especially when including input from other diverse populations.

## Abbreviations

T4W/Juntos: Together for Wellness/Juntos por Nuestro Bienestar

## Data Availability

The data sets generated during and analyzed during this study are available from the corresponding author on reasonable request.
